# 1.5 °C carbon budget dependent on carbon cycle uncertainty and future non-CO_2_ forcing

**DOI:** 10.1038/s41598-018-24241-1

**Published:** 2018-04-11

**Authors:** Nadine Mengis, Antti-Ilari Partanen, Jonathan Jalbert, H. Damon Matthews

**Affiliations:** 1 Concordia University, Montréal, Canada; 2 Finnish Meteorological Institute, Helsinki, Finland; 3 Ecole Polytechnique de Montréal, Montréal, Canada

## Abstract

Estimates of the 1.5 °C carbon budget vary widely among recent studies, emphasizing the need to better understand and quantify key sources of uncertainty. Here we quantify the impact of carbon cycle uncertainty and non-CO_2_ forcing on the 1.5 °C carbon budget in the context of a prescribed 1.5 °C temperature stabilization scenario. We use Bayes theorem to weight members of a perturbed parameter ensemble with varying land and ocean carbon uptake, to derive an estimate for the fossil fuel (FF) carbon budget of 469 PgC since 1850, with a 95% likelihood range of (411,528) PgC. CO_2_ emissions from land-use change (LUC) add about 230 PgC. Our best estimate of the total (FF + LUC) carbon budget for 1.5 °C is therefore 699 PgC, which corresponds to about 11 years of current emissions. Non-CO_2_ greenhouse gas and aerosol emissions represent equivalent cumulative CO_2_ emissions of about 510 PgC and −180 PgC for 1.5 °C, respectively. The increased LUC, high non-CO_2_ emissions and decreased aerosols in our scenario, cause the long-term FF carbon budget to decrease following temperature stabilization. In this scenario, negative emissions would be required to compensate not only for the increasing non-CO_2_ climate forcing, but also for the declining natural carbon sinks.

## Introduction

In the Paris Agreement, adopted on December 12^*th*^ 2015, 195 parties agreed to hold “the increase in the global average temperature to well below 2 °C above pre-industrial levels and to pursue efforts to limit the temperature increase to 1.5 °C above pre-industrial levels, recognizing that this would significantly reduce the risks and impacts of climate change” (Article 2 1.(a) of the Paris Agreement^1^). Using the now well-established finding of a linear climate response to cumulative carbon emissions (as measured by the Transient Climate Response to cumulative CO_2_ Emissions (TCRE)^2–4^), we can estimate the total allowable emissions associated with a 1.5 °C temperature target, the so-called 1.5 °C carbon budget. A robust estimate of the carbon budget for 1.5 °C has the potential to provide important information for current political discussions, surrounding the updating of future contributions towards emissions reductions from nations^5,6^.

More research has been dedicated to estimates of the 2 °C carbon budget (see review by Rogelj *et al*.^7^), than on the carbon budget associated with the more recently introduced 1.5 °C temperature target (see review by Matthews *et al*.^8^). Best estimates of the total 1.5 °C that exist vary widely, from as little as 600 PgC^9^ to more than 800 PgC^8,10^. Considering uncertainty ranges around these best estimates, increases the range of plausible budgets even further, from about 500 to 1100 PgC^11^. According to the Global Carbon Project, 555 ± 55 PgC of this budget has already been emitted by 2015 through fossil fuel (FF) and land-use-change (LUC) CO_2_ emissions^12^, which means that the range of allowable future emissions could extend from less than zero to as much as total historical CO_2_ emissions. Clearly, there is a critical need to better understand and constrain the uncertainty associated with 1.5 °C carbon budget estimates to produce an estimate that can usefully inform climate mitigation discussions.

Here, we present a probabilistic estimate of the 1.5 °C fossil fuel threshold avoidance carbon budget, from 1850 until the year at which 1.5 °C is reached, as well as for the 150 years of stable global mean temperature afterwards. In deriving this new budget estimate, we quantify the contribution of two key sources of uncertainty. First, we assess the contribution of uncertainty concerning the future carbon uptake capacity of the Earth system. About 48 ± 7% of current annual emissions are taken up by either the land or the ocean^12^, and these carbon sinks account for 57 ± 7% of cumulative emissions over the 1870–2015 period^12^. The future behavior of carbon sinks is a considerable source of variation among carbon budget estimates derived from different Earth-system models^8,10^. Second, carbon budget estimates depend strongly on scenarios of future non-CO_2_ emissions^7,8^. Here, we estimate the equivalent cumulative CO_2_ emissions represented by both historical and future non-CO_2_ forcing, which allows us to quantify their contribution to the historical budget and in addition highlights the considerable scenario uncertainty associated with future decisions about non-CO_2_ greenhouse gas mitigation.

## Results

### Probabilistic estimate of the historical and future CO_2_ carbon budgets

**Figure 1:**
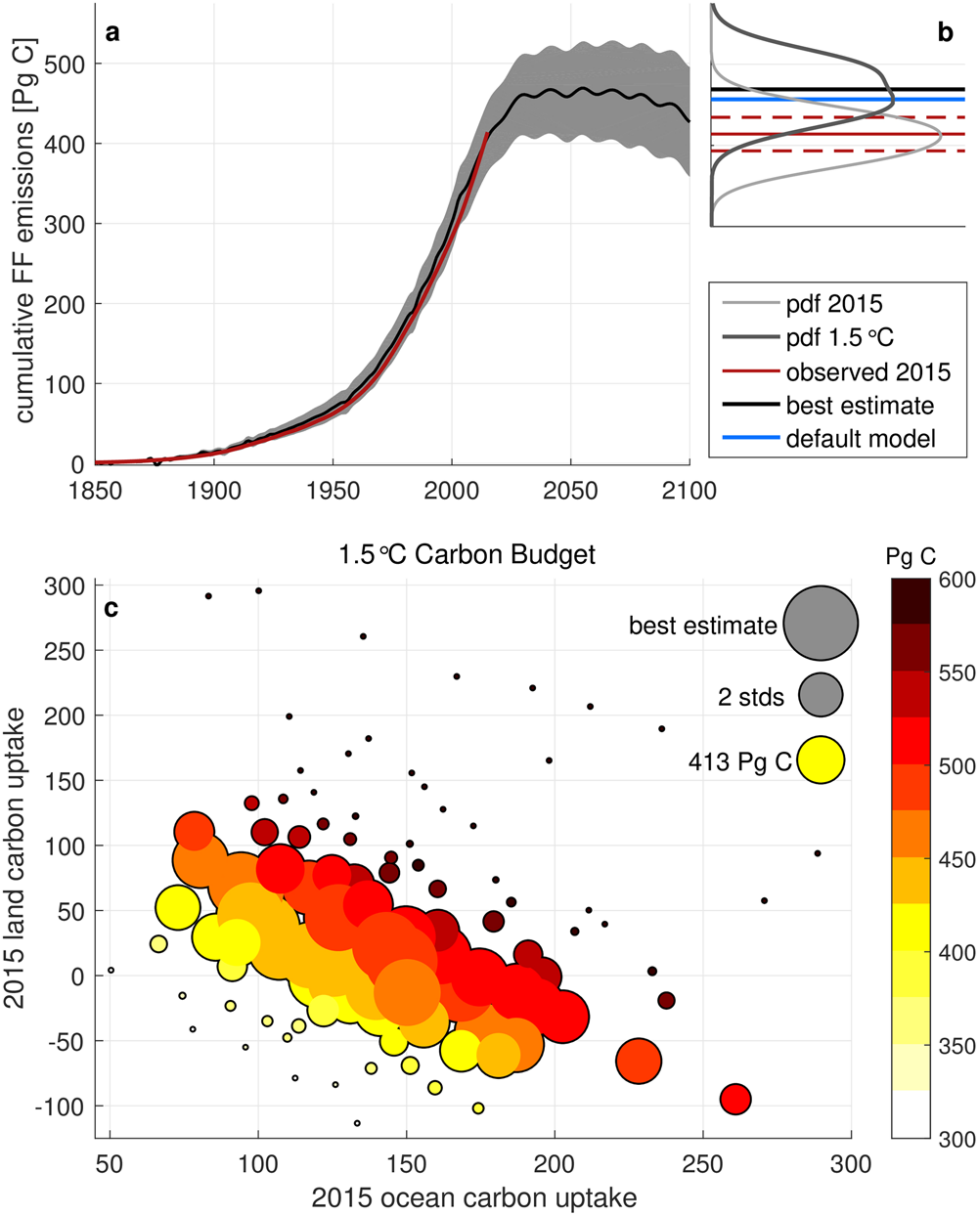
Probabilistic estimate of the fossil fuel only carbon budget. (**a**) Best estimate (black line) and 95% credible interval (light grey) of cumulative fossil fuel (FF) carbon emissions calculated from the perturbed parameter ensemble with varying ocean and land carbon uptake. Observed cumulative FF emissions (red line) for comparison. (**b**) Probability density of simulated cumulative FF emissions until 2015 (light grey) compared to the observed value of 413 PgC ±5% (red lines) given by the Global Carbon Cycle Project^12^ for the period of 1850–2015; Probability density of the 1.5 °C FF-only carbon budget (dark grey), with the best estimate value of 469 PgC (black line), compared to the default, i.e. un-manipulated, model simulation (blue line). (**c**) Estimates of the 1.5 °C FF-only carbon budget as a function of the 2015 cumulative ocean and land carbon uptake. Colours indicate the budget, while the size of the circle indicates the probability of the respective member of the perturbed parameter ensemble. Exemplary probability sizes are given for the best estimate, two standard deviations (2 stds) and the 2015 observed cumulative FF emissions (413 PG C)^12^.

Our best estimate of historical cumulative fossil fuel (FF) emissions is 410.5 PgC, with a standard deviation of 22.1 PgC, in good agreement with observations (413 ± 20.7 PgC^12^) (Fig. 1a and b). This estimate was produced using Bayes theorem to compare our perturbed parameter ensemble (PPE) of 100 simulations with co-varied land and ocean carbon uptake within an intermediate complexity Earth system model (University of Victoria Earth system climate model, UVic ESCM) with observed fossil fuel and cement emissions between 1850 and 2015 (see Methods). We then used this posterior distribution to weight the future model simulations to estimate the 1.5 °C carbon budget. The resulting probabilistic estimate is therefore constrained by cumulative historical FF emissions, accounting for prior knowledge of plausible carbon uptake parameters in the model, which are varied to reflect uncertainty associated with observed ocean and land carbon fluxes.

Our simulations suggest a FF-only carbon budget of 469 PgC with a 95% likelihood range of (411,528) PgC, which represents the total fossil fuel CO_2_ emissions since pre-industrial at the time that 1.5 °C is reached (around 2055 in our prescribed temperature scenario, see Fig. S1). The default (unperturbed) model, simulated an estimate of the 1.5 °C FF-only carbon budget of 457 PgC, which is close to the best estimate. The most likely 1.5 °C FF carbon budgets emerge from those combinations of ocean and land carbon uptake which are best able to reproduce observed carbon cycle behavior (Fig. 1c). Simulations with a high probability are located on a diagonal with a negative slope, in that they have a respectively high land carbon uptake combined with a respectively low ocean carbon uptake, or vice versa. In contrast, simulations with high land and a high ocean carbon uptake, and respectively simulations with a low land and low ocean carbon uptake are very unlikely.

**Figure 2:**
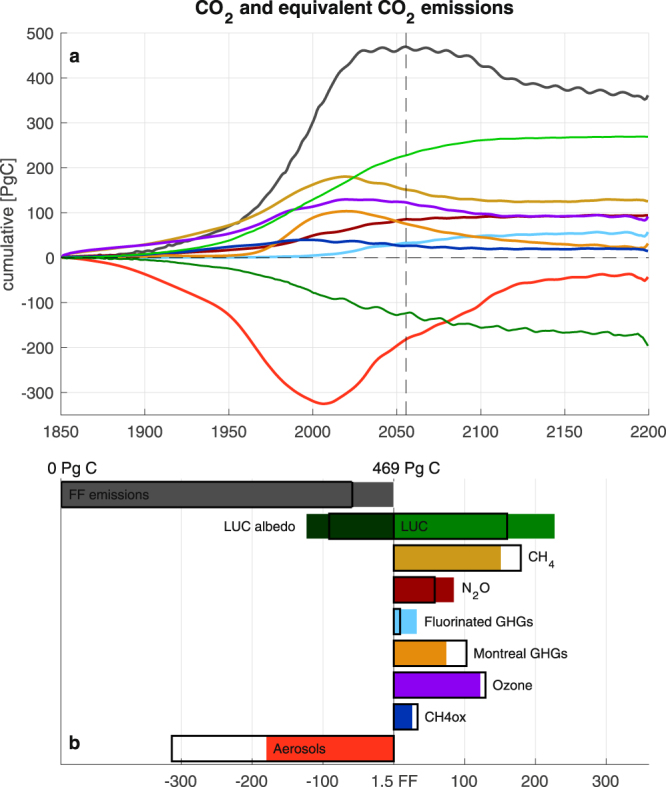
Equivalent CO_2_ emissions of non-fossil fuel climate forcing. (**a**) Best estimate of the cumulative fossil fuel (FF) emissions (grey) and equivalent CO_2_ emissions from non-FF forcing derived with the default model as a function of time (see **b** for colour legend). (**b**) Contributions from fossil fuel (FF) and non-FF forcing to the 1.5 °C carbon budget. The filled bars give the values for the total contribution between 1850 and 1.5 °C, and the black boxes show the values for the historical contribution (1850–2015). Land-use changes (LUC) accordingly contributed 160 PgC in the historical period (black box) and about 230 PgC for the total 1.5 °C budget.

This probabilistic carbon budget estimate applies only to FF emissions, since in our model set-up, spatial land-use changes (LUC) were prescribed, and the emissions produced by LUCs were therefore included as part of the net land carbon uptake used to constrain the model simulations. LUC emissions in the default model amounted to 160 PgC up to 2015 (Fig. 2a) (in good agreement with observed LUC emissions of 158 PgC^12^). As a result of the relatively large expansion of LUC in RCP2.6^13^, cumulative LUC emissions in our simulations increased by 70 PgC between 2015 and 2055, reaching a total contribution of 230 PgC at year 2055. Our best estimate of the total (FF + LUC) carbon budget for 1.5 °C is therefore 699 PgC. Subtracting 570 PgC of simulated historical emissions until 2015 gives a remaining total carbon budget of 129 PgC. This represents about 11 years of current FF + LUC CO_2_ emissions.

### Contributions of non-CO_2_ forcing to the historical and 1.5 °C budgets

Our carbon budget estimate and its uncertainty is constrained by the observed carbon cycle behaviour, but is also contingent on our chosen scenario of non-CO_2_ emissions. When quantified as equivalent cumulative CO_2_ emissions, the effect of non-CO_2_ greenhouse gases (GHGs) and aerosol emissions are both substantial (Fig. 2). This demonstrates that estimates of future carbon budgets are highly sensitive to non-CO_2_ mitigation decisions.

The cumulative equivalent CO_2_ emissions of the main non-CO_2_ GHGs (methane (CH4), nitrous oxide (N2O), fluorinated GHGs and GHGs covered by the Montreal Protocol) for the historical period amount to a total of about 350 PgC in 2015 (Fig. 2a). This estimate is almost as high as the best estimate of the historical FF emissions in 2015 (410.5 PgC). In addition to these, the equivalent CO_2_ emissions of the tropospheric and stratospheric ozone forcing and the methane oxidation forcing contribute about 130 PgC and 35 PgC, respectively. These positive equivalent CO_2_ emissions are partly compensated by the negative equivalent CO_2_ emissions from the direct and indirect forcing of aerosols, which amount to about −315 PgC in 2015. Compared to the relation of cumulative CO_2_ emissions to induced radiative forcing from CO_2_, the magnitudes of these cumulative equivalent CO_2_ emissions of non-CO_2_ climate forcing agents agree well with the magnitudes of their respective observed radiative forcing^11^.

**Figure 3:**
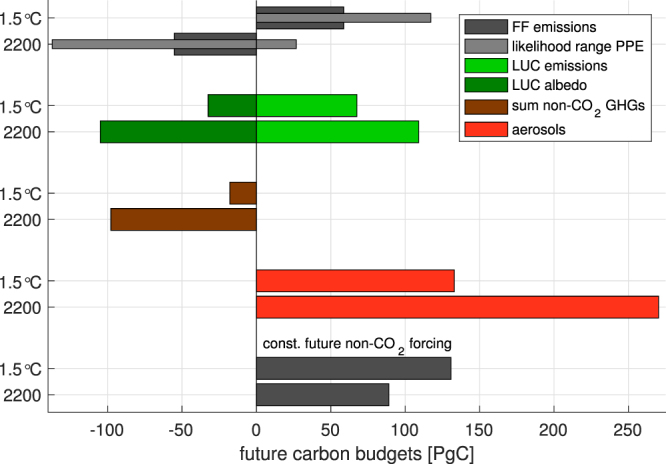
Future carbon budgets. The future carbon budgets for both i) the 1.5 °C target and ii) the 2200 long-term stabilization temperature target relative to 2015. The first panel gives the best estimate for the fossil fuel (FF) emissions, including the uncertainty range obtained from the perturbed parameter ensemble (PPE). The best estimate remaining FF 1.5 °C budget since 2015 is 60 PgC, while the long term future budget until 2200 is −55 PgC. The other panels show the same estimates for land-use change (LUC), as well as equivalent CO_2_ emissions from non-CO_2_ GHGs and aerosols. The lowest panel shows future FF emissions in case of constant 2015 non-CO_2_ GHGs and aerosol forcing.

Given the large contributions of both positive and negative non-CO_2_ forcing at 2015, our estimate of the future cumulative CO_2_ emissions for 1.5 °C depends on the balance of these two contributions after the year 2015. In our scenario (based on RCP2.6 non-CO_2_ emissions; see Methods) non-CO_2_ GHGs maintain their high contribution to global warming; the carbon budget equivalent of all non-CO_2_ GHG forcing (including ozone and methane oxidation) decreased from 510 PgC in 2015 to 495 PgC at 1.5 °C (a decreased contribution of only 15 PgC). In contrast, the carbon budget contribution of aerosols decreased substantially, from −315 PgC in 2015 to −180 PgC at 1.5 °C. Therefore, the sum of the future contributions of non-CO_2_ forcing agents decreased our estimate of the 1.5 °C carbon budget by 135 − 15 = 120 PgC (see upper bars of Fig. 3). In an alternate scenario whereby we held non-CO_2_ forcing constant after 2015, we hence found the 1.5 °C carbon budget with 130 PgC to be higher than for the simulation with changing non-CO_2_ emissions (Fig. 3).

### Temperature stabilization carbon budget

The effect of positive net non-CO_2_ forcing also had a large effect on the evolution of the simulated carbon budget after temperatures stabilized in our simulations, with the result that the long-term (year 2200) FF carbon budget was considerably smaller than the budget at the time that 1.5 °C was reached (Fig. 3). At 2200, our best estimate of the FF carbon budget decreased to 355 (274, 441) PgC, which represents a decrease of 112 PgC from the FF-only budget at the year 2055, and a decrease of 53 PgC relative to cumulative FF emissions up to the year 2015 (Fig. 3). During this temperature stabilization period, continued LUC in our scenario resulted in an additional 40 PgC of emissions. This represents a total long-term budget of 625 PgC to maintain temperatures at 1.5 °C until the year 2200, compared to 699 PgC at the 2055.

Most of this decrease is driven by a continued reduction in aerosol forcing after the year 2055, which increased the carbon budget equivalence of changing aerosol forcing (relative to 2015) from 130 PgC at 2055 to 275 PgC at the year 2200. The effect of decreased non-CO_2_ greenhouse gas forcing was considerably smaller, representing an increased allowance for CO_2_ emissions from 15 PgC at 2055 to only 100 PgC at 2200. The net effect of non-CO_2_ forcing in our scenario was therefore to reduce the available carbon budget by 60 PgC between 2055 and 2200.

## Discussion

In this study, we have provided a new best-estimate and likelihood range for the fossil fuel (FF) carbon budget that is constrained by cumulative historical FF emissions, and accounts for prior knowledge about the historical ocean and land carbon fluxes. Our resulting 1.5 °C budget estimates for FF-only CO_2_ emissions are 469 (411,528) PgC at 2055 (the year temperature change reaches 1.5 °C), and 355 (274,441) PgC at the year 2200 (after 145 years of prescribed global mean temperature change of 1.5 °C). Including CO_2_ emissions from land-use change increases the best-estimate budget to 699 GtC at 2055 and 625 PgC at 2200. Given the large contribution of LUC to future CO_2_ emissions in our model, these results suggest that curtailing deforestation would be an effective way of increasing the allowable FF carbon budget.

However, even after accounting for the LUC contribution, our budget estimates are considerably smaller than some recently published 1.5 °C budgets^8,10^. Much of this difference relates to the time-evolution of non-CO_2_ forcing agents in our study; in particular, the aerosol forcing (derived from spatial patterns of optical depth based on RCP2.6 aerosol emissions) declines faster than the globally-averaged aerosol forcing used by Millar *et al*.^10^. In the simulation where we held non-CO_2_ forcing constant at present-day levels, the allowable FF budget was substantially larger (about 520 and 470 PgC at 2055 and 2200, respectively), and when including LUC emissions, the total budget in this scenario was close to 745 PgC at both 2055 and 2200.

A critical question that emerges from our results is to what extent negative emissions would be required to stabilize temperatures at 1.5 °C once this level of warming is reached. One insight that emerges from the carbon cycle dynamics in our model is that stable global temperatures requires declining atmospheric CO_2_ concentrations in the presence of residual non-CO_2_ forcing, which rapidly negates the land carbon sink, and greatly slows carbon uptake by the ocean. In fact, by the year 2070 in our simulations, the combined land and ocean carbon uptake decreased to zero and the carbon cycle began to release previously stored carbon back to the atmosphere (Fig. S2). Our base scenario suggests that substantial quantities of negative emissions would therefore be required to counter this loss of natural carbon sinks. However, it is important to emphasize that this conclusion is specific to the non-CO_2_ forcing scenario, where the net non-CO_2_ (greenhouse gas and aerosol) forcing continued to increase. Most of this increase reflects declining aerosol emissions, which would clearly have benefits to human health^14^, but also further constrain the allowable carbon emissions for the 1.5 °C climate target.

Our results therefore carry several key policy implications. First, by constraining climate model simulations using observational carbon cycle uncertainty we were able to narrow the uncertainty associated with future carbon budgets, and given the small budget associated with the 1.5 °C temperature target, we are not able to exclude the possibility that we have already exceeded the remaining carbon budget for this target. Second, even in the case of a non-zero future carbon budget, maintaining temperature change at 1.5 °C without resorting to negative CO_2_ emissions and while reducing the atmospheric aerosol burden, will likely require more stringent mitigation of non-CO_2_ greenhouse gas emissions than what is represented by even the most ambitious current mitigation scenarios.

## Methods

### Model description

For our study we used version 2.9 of the University of Victoria Earth System Climate Model (UVic ESCM), a climate model of intermediate complexity. It includes schemes for ocean physics based on the Modular Ocean Model Version 2 (MOM2)^15^, ocean biogeochemistry^16^, and a terrestrial component including soil and vegetation dynamics represented by 5 plant functional types^17^. The atmosphere is represented by a two dimensional atmospheric energy moisture balance model, including a thermodynamic sea ice model^18,19^. All model components have a common horizontal resolution of 3.60° longitude and 1.8° latitude and the oceanic component has a vertical resolution of 19 levels, with vertical thickness varying between 50 m near the surface to 500 m in the deep ocean. The UVic ESCM is a well established Earth system model with a good evaluation of its carbon cycle processes^20^.

### Scenario design and diagnosed emissions

For our simulations we have prescribed a temperature change scenario as the input to the UVic ESCM, and used the model to estimate the fossil fuel CO_2_ emissions trajectory that is consistent with this temperature trajectory, as in Zickfeld *et al*.^3^ and Matthews *et al*.^21^. When running the model in this mode, atmospheric CO_2_ concentrations are adjusted dynamically by the model so as to achieve the prescribed temperature change, and the consistent CO_2_ emissions are diagnosed as a function of simulated atmospheric CO_2_ as well as land and ocean carbon sinks. Our prescribed temperature scenario followed the model-simulated temperature response to historical forcing up to the year 2015, and then approached 1.5 °C above 1850–1879 temperature at about the year 2055 (Fig. S1).

Future non-CO_2_ forcing, as well as the spatial distribution of land-use changes are based on the representative concentration pathway 2.6 (RCP2.6), which is an ambitious mitigation scenario^13^. Non-CO_2_ greenhouse gas forcing is taken directly from RCP2.6. However, rather than using the global-average RCP2.6 aerosol forcing from the RCP database, we instead used RCP2.6 spatially explicit patterns of aerosol optical depth (AOD), which decrease almost linearly after the year 2050. Both the simulated year-2000 and the year-2100 RCP2.6 anthropogenic aerosol forcing in the UVic ESCM compare well with the anthropogenic aerosol forcing reported with comprehensive aerosol-climate models for the given scenario^22^. After 2100, we continued this linear decrease until AOD reached zero at the year 2112. This is based on the assumption that even further measures would be taken after 2100 to reduce air pollution, to minimize negative health impacts caused by anthropogenic aerosol emissions^23^.

### Perturbed Parameter Ensemble (PPE)

To assess uncertainties in the land and ocean carbon uptake we increased or decreased land and ocean carbon uptake in the model by adjusting the level of atmospheric CO_2_ that was used by the model components carbon cycle to estimate the rate of carbon uptake. This allowed us to scale the land and ocean carbon uptake among our 100 ensemble members. We chose a range of parameters that is in agreement with the a priori probability of observed land and ocean carbon fluxes^12^ (Fig. S3a).

### Probabilistic estimate of the carbon budget using Bayes Theorem

From the perturbed parameter ensemble obtained by varying the land and ocean carbon uptake parameters, we computed the posterior probability of each member according to the observed cumulative carbon emissions between 1850 and 2015. The observations were provided by the Global Carbon Cycle Project^12^. The ensemble member *i* ∈ 1, …, 100 denoted by ${ {\mathcal M} }_{i}$, is characterized by its predicted cumulative emission *x*_*i*_ between 1850 and 2015 and its land and ocean carbon uptake parameters (*a*_*i*_, *b*_*i*_). Let *Y* denote the observed carbon emissions. We want to weight each member $({ {\mathcal M} }_{i}:i=1,\ldots ,100)$ according to its capability to represent the observed carbon emission. The statistical model for achieving this is the following:

1 $$Y={x}_{i}+\varepsilon ,i=1,\ldots ,100;$$

where $\varepsilon \sim {\mathscr{N}}(0,{20.63}^{2})$, represent the uncertainty on observations given by the Global Carbon Cycle Project. The importance (the weight) of the member ${ {\mathcal M} }_{i}$ for predicting the observed value is computed using Bayes’ theorem:

2 $${\mathbb{P}}({ {\mathcal M} }_{i}|Y=\mathrm{413)}\propto {\mathscr{N}}\mathrm{(413}|{x}_{i},{20.63}^{2})\times {\mathbb{P}}({ {\mathcal M} }_{i});$$

where ${\mathscr{N}}(y|\mu ,{\sigma }^{2})$ denotes the normal density with parameters (*μ*, *σ*^2^) evaluated at *y* and ${\mathbb{P}}({ {\mathcal M} }_{i})$ corresponds to the prior importance of member ${ {\mathcal M} }_{i}$. The prior probability can be computed by matching the outcome of model simulations with independent land and ocean parameter perturbations to the observed cumulative land and ocean carbon uptake between 1959 and 2015. The slightly non-linear scaling of the parameter input to the model output, causes the normal distributions given from the observational data to be transformed into log-normal distributions (Fig. S3a). Note, that the posterior probabilities (Fig. S3b) are not sensitive to this prior choice. We verified it by putting an equal prior weight of 1/100 to all members, which corresponds to a non-informative prior. The resulting estimation of the 2015 carbon budget was almost the same (Fig. S4). The informative priors ensure though that an impossible combination of carbon uptake parameters would never be considered, even if its cumulative emission is in the range of the observations.

### Estimate of equivalent CO_2_ emissions of non-CO_2_ climate forcing and land-use change (LUC) emissions

To estimate the cumulative CO_2_ emissions that are equivalent to a given non-CO_2_ forcing, the default (i.e. unperturbed) UVic ESCM was forced to follow the same temperature trajectory, while removing each individual forcing from the model input (i.e. the radiative forcing of an individual greenhouse gas (GHG), land-use changes or spatially resolved aerosol forcing). To follow the temperature trajectory, the model needed to adjust the diagnosed CO_2_ emissions to account for the missing input forcing. The difference between the all-forced and the reduced-forced diagnosed cumulative CO_2_ emissions represents the equivalent cumulative CO_2_ emissions of the respective non-CO_2_ forcing. In case of the equivalent CO_2_ emissions for land-use change (LUC) these emissions account for the carbon emissions from LUC as well as the albedo changes from the transformed land surface.

To separate these two effects, we performed an additional simulation to estimate carbon emission from LUC only. In this second simulation we apply constant pre-industrial land-use conditions, but instead of tracking atmospheric temperature we prescribe the same CO_2_ concentration increase from the changing land-use simulation. The difference between the total land carbon content of the no-LUC simulation and the changing-LUC simulation represents the LUC carbon emissions^24^. The equivalent CO_2_ emissions form LUC induces albedo changes is accordingly the difference between the previously calculates equivalent CO_2_ emissions from the total LUC forcing (emissions and albedo), and the separately determined LUC carbon emissions.

### Data availability

The data that support the findings of this study are available from the corresponding author upon reasonable request.

## Electronic supplementary material

Supporting Information
