# Supplementary material for: 1.5 °C carbon budget dependent on carbon cycle uncertainty and future non-CO2 forcing
Source: Sci Rep. 2018 Apr 11;8:5831. doi: 10.1038/s41598-018-24241-1 (PMC5895820; doi:10.1038/s41598-018-24241-1)
Supplement: Supplementary file 1 — Supporting Information [file 41598_2018_24241_MOESM1_ESM.pdf]

Supporting Information: 1.5°C carbon budget dependent on carbon cycle uncertainty and future non-CO_2 forcing

Nadine Mengis[1,*], Antti-Ilari Partanen[1,2], Jonathan Jalbert[3], and H. Damon Matthews[1]

[1]Concordia University, Canada
[2]Finnish Meteorological Institute, Finland
[3]Ecole Polytechnique de Montréal, Canada
[*]nadine.mengis@concordia.ca

ABSTRACT

Estimates of the 1.5°C carbon budget vary widely among recent studies, emphasizing the need to better understand and quantify key sources of uncertainty. Here we quantify the impact of carbon cycle uncertainty and non-CO_2 forcing on the 1.5°C carbon budget in the context of a prescribed 1.5°C temperature stabilization scenario. We use Bayes theorem to weight members of a perturbed parameter ensemble with varying land and ocean carbon uptake, to derive an estimate for the fossil fuel (FF) carbon budget of 469 PgC since 1850, with a 95% likelihood range of (411,528) PgC. CO_2 emissions from land-use change (LUC) add 228 PgC. Our best estimate of the total (FF+LUC) carbon budget for 1.5°C is therefore 697 PgC, which corresponds to about 11 years of current emissions. Non-CO_2 greenhouse gas and aerosol emissions represent equivalent cumulative CO_2 emissions of 512 PgC and -180 PgC for 1.5°C, respectively. The increased LUC, high non-CO_2 emissions and decreased aerosols in our scenario, cause the long-term FF carbon budget to decrease following temperature stabilization. In this scenario, negative emissions would be required to compensate not only for the positive non-CO_2 climate forcing, but also for the declining natural carbon sinks.

Temperature trajectory for the threshold avoidance budget

To obtain a so-called threshold or temperature avoidance budget for the ΔT=1.5°C target, we prescribed a surface air temperature trajectory, and ran the model in a mode, in which it diagnosed the necessary fossil fuel (FF) emission to reach this temperature target. This way all members of the perturber parameter ensemble do follow the same temperature trajectory (Fig. S1), but have varying ocean and land carbon uptake (Fig. S2).

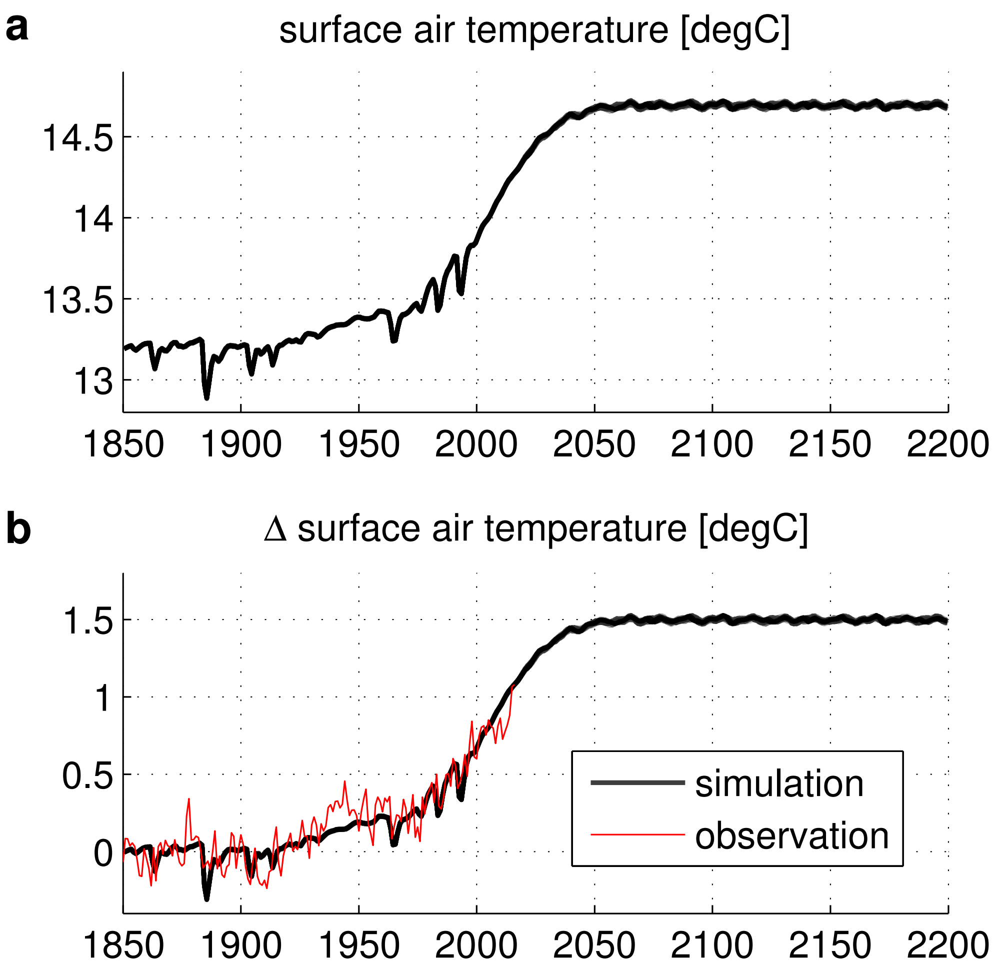

Figure S1. Temperature trajectory for the 1.5°C temperature change
a: Temperature output from the 100 members of the perturbed parameter ensemble **b**: as temperature anomaly from the 1850-1879 reference period. The HadCRUT4-gl data reference to the same pre-industrial period is shown for comparisons (red line)[?].

Land and ocean carbon fluxes

The temporal development of the total land and ocean carbon flux shows the transition from a total carbon sink of the natural system to a carbon source in the later part of the 21st century (Fig. S2a). Considering the single earth system components it becomes evident, that while the ocean carbon uptake is reduced over this period of time (Fig. S2b), the actual release of carbon from the natural system is driven by the land reservoir (Fig. S2c).

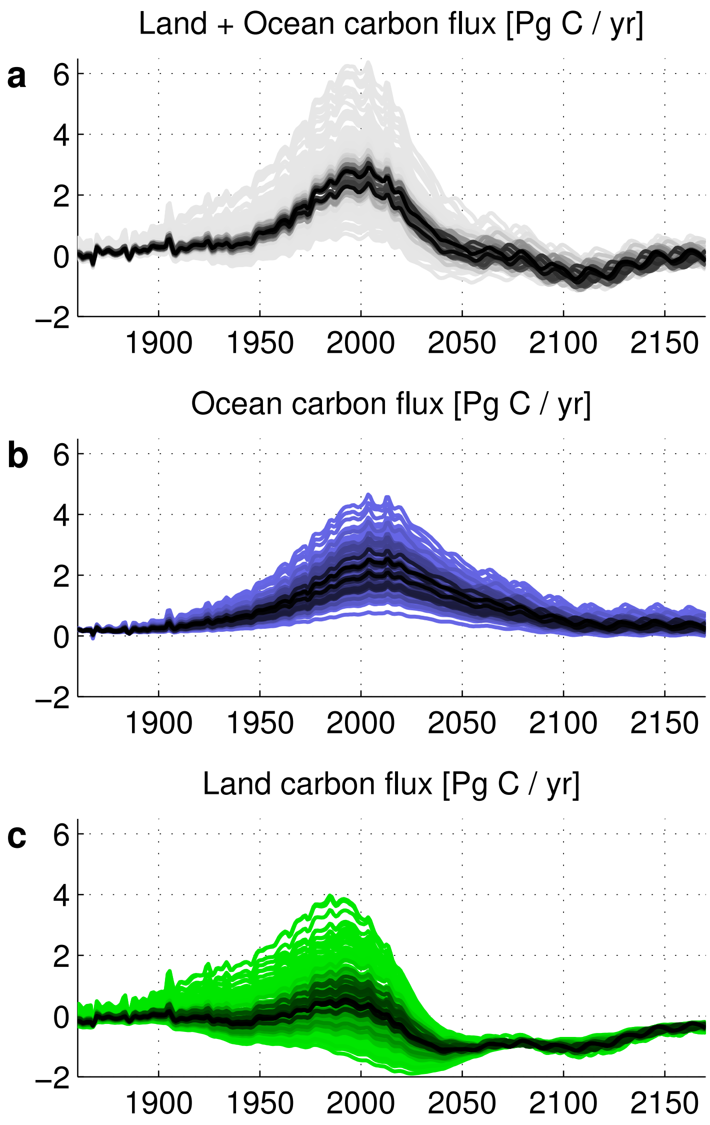

Figure S2. Land and ocean carbon fluxes
Time series of ocean+land (**a**), ocean (**b**) and land (**c**) from all 100 members of the perturbed parameter ensemble, shading indicates the likelihood of the ensemble member given the posterior distribution. Note, that a 20 years running mean was applied to smooth the single members.

A priori and posterior weights for the perturbed parameter ensemble

Varying the model input parameters to manipulate the land and ocean carbon fluxes, results in varying historical (1959-2015) cumulative ocean and land carbon uptake. Taken the uncertainty of these observed fluxes[?] into account, the a priori weights for the single members were estimated (Fig. S3a). Using Bayes theorem to constrain the simulated fossil fuel emissions by the observed values[?] provides the posterior weights (Fig. S3b).

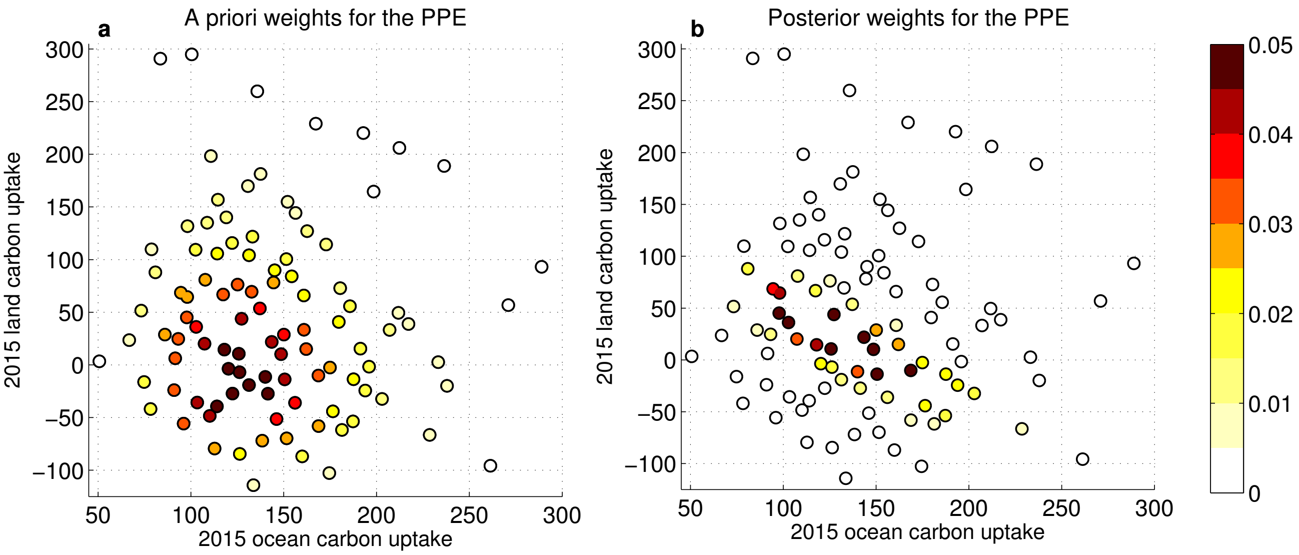

Figure S3. Scatter plots of the a priori and posterior weights of the simulations
A priori (**a**) and posterior (**b**) weights of the 100 members of the perturbed parameter ensemble (PPE), as a function of 1959-2015 cumulative ocean and land carbon fluxes.

Effect of a priori distribution on the probability density of the 2015 carbon budget

In our study we apply the a priori weights shown in figure S3a to the estimate of the posterior weights shown in figure S3b. The probability density for the 1850 to 2015 carbon budget shown in figure 1b of the main article is the result of these posterior weights. Here we show the influence of the a priori distribution to this estimate, by performing the same calculations but using a flat a priori distribution instead, meaning that all ensemble members have equal weight.

We find that the a priori distribution does not have a strong influence on the posterior probability density function of the 1850-2015 cumulative carbon emissions (Fig. S4). The distribution with a flat prior has a slightly higher mean value by about 5 Pg C, and the distribution has a slightly smaller variance, but the general shape remains the same.

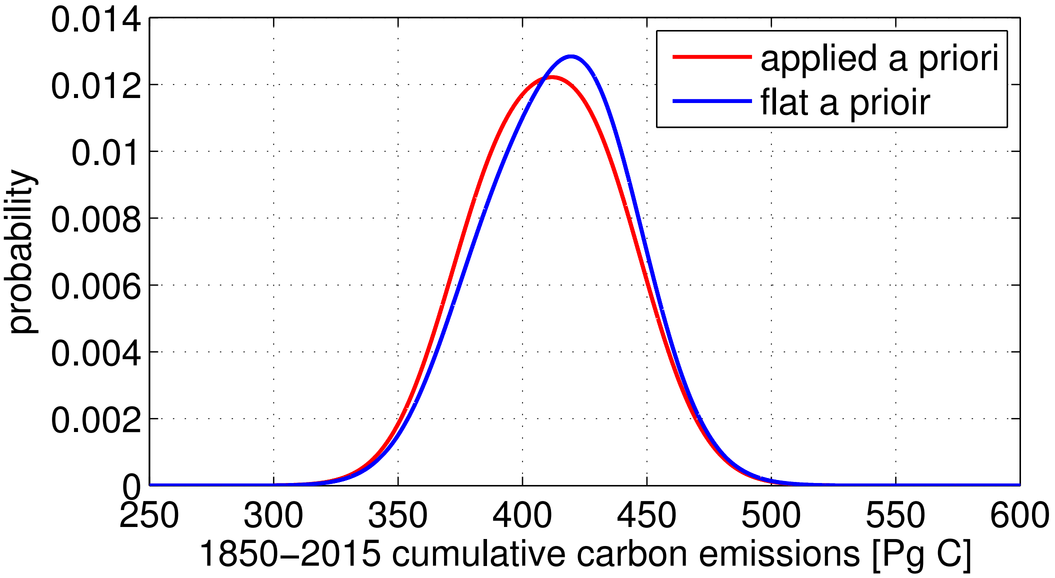

Figure S4. Effect of a priori distribution on the probability density of the 2015 carbon budget
The posterior probability density function of the historical carbon budget, used for the weighting of the future carbon budgets. (red line): As used in this study with prior information on the probability of ocean and land input parameters, and (blue line): with the assumption of no prior knowledge of the likelihood of input parameters, i.e. with a flat a priori distribution.
